# Artificial Intelligence Enhances Studies on Inflammatory Bowel Disease

**DOI:** 10.3389/fbioe.2021.635764

**Published:** 2021-07-08

**Authors:** Guihua Chen, Jun Shen

**Affiliations:** Division of Gastroenterology and Hepatology, Key Laboratory of Gastroenterology and Hepatology, Ministry of Health, Inflammatory Bowel Disease Research Center, Shanghai Institute of Digestive Disease, Renji Hospital, School of Medicine, Shanghai Jiao Tong University, Shanghai, China

**Keywords:** inflammatory bowel disease, artificial intelligence, Crohn’s disease, ulcerative colitis, machine learning

## Abstract

Inflammatory bowel disease (IBD), which includes ulcerative colitis (UC) and Crohn’s disease (CD), is an idiopathic condition related to a dysregulated immune response to commensal intestinal microflora in a genetically susceptible host. As a global disease, the morbidity of IBD reached a rate of 84.3 per 100,000 persons and reflected a continued gradual upward trajectory. The medical cost of IBD is also notably extremely high. For example, in Europe, it has €3,500 in CD and €2,000 in UC per patient per year, respectively. In addition, taking into account the work productivity loss and the reduced quality of life, the indirect costs are incalculable. In modern times, the diagnosis of IBD is still a subjective judgment based on laboratory tests and medical images. Its early diagnosis and intervention is therefore a challenging goal and also the key to control its progression. Artificial intelligence (AI)-assisted diagnosis and prognosis prediction has proven effective in many fields including gastroenterology. In this study, support vector machines were utilized to distinguish the significant features in IBD. As a result, the reliability of IBD diagnosis due to its impressive performance in classifying and addressing region problems was improved. Convolutional neural networks are advanced image processing algorithms that are currently in existence. Digestive endoscopic images can therefore be better understood by automatically detecting and classifying lesions. This study aims to summarize AI application in the area of IBD, objectively evaluate the performance of these methods, and ultimately understand the algorithm–dataset combination in the studies.

## Introduction

Inflammatory bowel disease (IBD) is a chronic inflammatory disorder with soaring incidences recorded worldwide in recent years. For example, in Western countries, the prevalence of IBD has exceeded 0.3% and is expected to continue rising steadily over the next decade. In the same light, several studies have drawn attention to the similarity between the current prevalence of IBD in newly industrialized countries, with anterior patterns observed in the Western world. The global burden on the healthcare system caused by IBD is also currently increasingly becoming a high-priority matter (Windsor and Kaplan, 2019). It is important to note that the complete etiology of IBD is still uncertain. However, the two major manifestations of IBD, namely, ulcerative colitis (UC) and Crohn’s disease (CD), are recognized as a result of complex interactions between genetic and environmental factors. A recent genetic association study identified 163 susceptibility loci for IBD. In this case, the impact of host–microbe interactions in pathogenesis was scrutinized due to the link between these susceptibility loci and the microbial response (Liu et al., 2015). The intestinal microecology has also been a focal point for recent studies (Nishida et al., 2018; Yue et al., 2019).

Currently, there is no clearly defined criterion for diagnosing IBD (Annese et al., 2013; Magro et al., 2013). Laboratory tests that include blood, stool, serum markers, and gene examination can be supportive in estimating the severity of IBD but are unable to confirm the diagnosis. As a fundamental process in the diagnosis of suspected patients with IBD, an endoscopic examination improves the accuracy of the diagnosis. On the other hand, biopsies of selected bowel segments can further confirm the diagnosis and make a valid assessment of the state of the IBD and its progression. However, it has been observed that endoscopic images and histological changes are evident only when the appropriate sampling sites are selected, and the images are of good quality. The undifferentiated results were obtained in some of the patients, which in turn resulted in the delayed diagnosis or reclassification over time, proving that the definite diagnosis of IBD is still a challenging endeavor (Negreanu et al., 2019).

**FIGURE 1:**
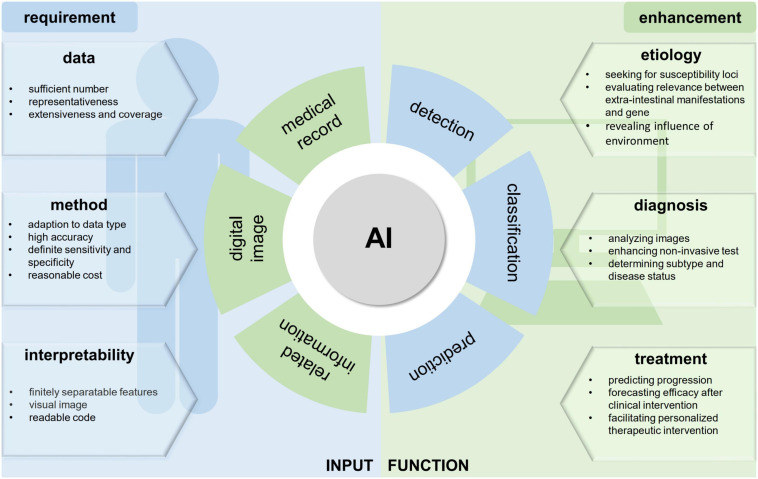
The requirement of artificial intelligence (AI) application and the enhancement for inflammatory bowel disease (IBD) studies. The high-quality data at a certain volume are the basis of building and training the machine learning (ML) models. Making the appropriate match between the dataset and analysis methods is also an essential step, which can influence both accuracy and interpretability of results. The AI application in IBD study was summarized and sorted by the research purpose. In the field of IBD etiology, diagnosis, and treatment, AI methods played distinct roles.

The goal of treatment of IBD has evolved from traditional clinical remission to a more specific, integrated, and complete deep remission or mucosal healing (Panaccione et al., 2013; Sandborn et al., 2014; Klenske et al., 2019). And the advent of biological agents, such as anti-adhesion molecules, anti-cytokine molecules, blockage of downstream signaling, and anti-trafficking molecules promoted the achievement of goal in the treatment of IBD. Intestinal microbiome manipulation and stem cell transplantation are also the treatment option for certain patients with IBD (Na and Moon, 2019). Considering the development of research about IBD, its combination with artificial intelligence (AI) technology is not simply a result of interdisciplinary thinking but an inevitable merging of the two treatments. Digital images and medical records require advanced technology as this would lead to a great extent to significant strides being made in the studies of IBD (Figure 1).

## Artificial Intelligence

Artificial intelligence, in particular, the deep-learning subtype has emerged as a breakthrough in computer technology enabled by the application of labeled big data across all the sectors (Topol, 2019). AI allows computers to identify, quantify, and interpret relationships among variables by algorithmically learning the efficient data representations, which is a formidable task for physicians (Johnson et al., 2018). As a technique being initially confined as “the science and engineering of making intelligent machines,” AI was considered in medical research when its ability to learn complex patterns and make predictions was noticed. From the 1950s to present, machine learning (ML) has become the most common approach of AI mostly owing to the ML models with the function of prediction or making decisions including support vector machines (SVMs), neural network, Naïve Bayes (NB), and random forest (RF).

**FIGURE 2:**
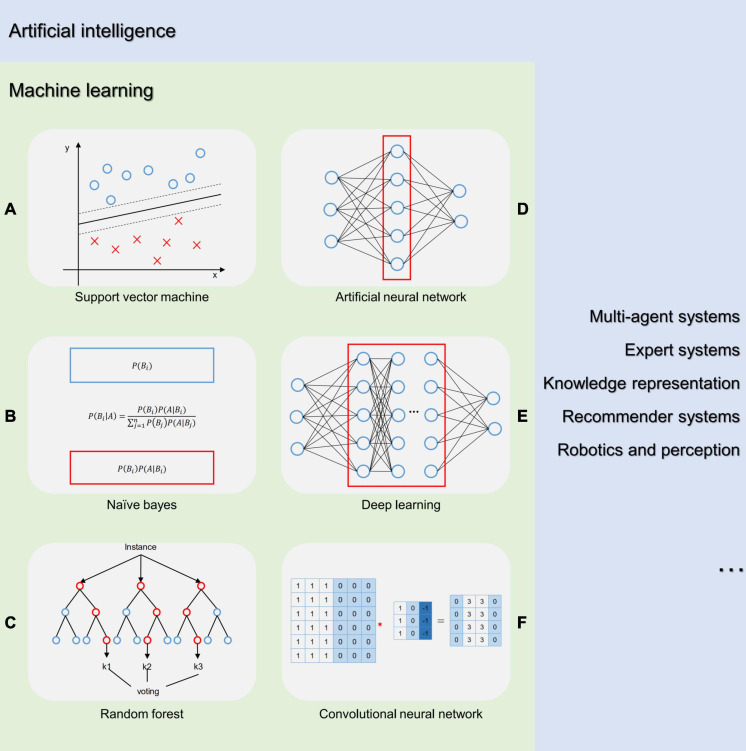
Artificial intelligence terminology. AI is a general term concerning the computer science that enables the machine applying the human intelligence such as “learning” and “problem-solving” to perform the practical task. ML, multi-agent systems, expert systems, knowledge representation, recommender systems, robotics, and perception are the subset of AI. ML is a subset of AI, which automatically detects patterns in data to make predictions or decisions without explicitly being programmed. **(A)** Support vector machine (SVM) is a discriminative classifier that has an excellent performance in classification due to its strength of regularization and convex optimization. With the application of “kernel trick,” SVM can handle non-linear problems. **(B)** Naïve Bayes is a probabilistic ML algorithm based on the Bayes’ theorem and assumes the independence between features. **(C)** Random forest is an ensemble algorithm constructing numerous decision trees at training to increase the overall result by combining learning models. **(D)** Artificial neural network (ANN) is a regression and classification algorithm composed of artificial neurons applying simple classifiers to output decision signals based on the weighted sum of evidence. The basic structure was the combination of an input, hidden connection, and output layer. **(E)** Deep learning is a class of ANNs with several hidden layers which can learn complex hierarchical representations from the data for feature extraction and transformation, and for pattern analysis and classification. **(F)** Convolutional neural network (CNN) is a specific subset of ANNs that imitate the organization of the animal visual cortex for image processing tasks. The convolutional layer of CNN is the essential part.

Artificial neural network (ANN), a remarkable one of these AI approaches, was structured with an input, hidden connection, and output layer. The basic computing units of ANN are the simulation of neurons and synapses in the human brain and are responsible for outputting a decision signal based on the weighted sum of evidence. Deep learning (DL) was developed based on ANN with extra hidden layers between the input and output layers to overcome its shortcomings such as overfitting, vanishing gradient, and decreasing in the local minimum during optimization. It impressed the researchers due to the excellent performance of convolutional neural networks (CNNs) in the computer vision field. The outstanding performance depended on the preprocessing operation named convolution that applied specific filters to draw specific features and created numerous feature maps. Then, the feature maps were handled by the pooling layers to smaller sizes, and this process of convolutional and pooling layers was duplicated. The fully connected layers were the last step to produce the combination of all features and make an overall classification (Figure 2).

## Artificial Intelligence in the Etiology of IBD

In recent years, the etiology of IBD has expanded from a focus on abnormal gene expression to more complex factors such as the environmental and intestinal microflora (Piovani et al., 2019). However, genetic susceptibility continues to seek breakthroughs, and many results are available (Momozawa et al., 2018). AI facilitates the data analysis used to determine suspicious risk genes, which are then verified experimentally. However, AI does not seem to be widely used in intestinal microecology due to the current lack of adequate and uniform data records. Considering the novel perspectives on the pathogenesis of IBD in the study of intestinal microecology and the demand for a large amount of data processing, AI technology will be applied to the study of IBD pathogenesis by intestinal flora.

### Seeking IBD-Associated Susceptibility Loci and Genes

In the past, genome-wide association studies (GWAS) were used to analyze the genome of patients for identifying possible disease-related sequence variations (Liu et al., 2015; Luo et al., 2017). The principle of GWAS was to compare the allele frequencies of a specific variant between unrelated cases and controls to test significant differences that are expected to be disease-related (Ye and McGovern, 2016). So far, around 200 genes implicated in the etiology of IBD have been found through GWAS and differential gene expression analysis (Jostins et al., 2012). Although more genes are expected to participate in the pathophysiological process (Khor et al., 2011), the existing study methods are incompetent to mine the massive and complex genomic data utilizing only visual investigation of pairwise correlations. Moreover, the effectiveness of GWAS was limited by its nature as a non-candidate-gene approach leading to a high occurrence of false-positive results. The associated single nucleotide polymorphisms (SNPs) identified by GWAS are not usually the direct causal ones with disease because of the case-control designed only to reveal associations but not causation (Du et al., 2012). Instead, analytical tools are in demand to support the discovery of unforeseen relationships, to develop new hypotheses or models, and to forecast. The design of ML algorithms automatically detects patterns in data and accordingly fits the data-driven sciences especially genomics (Eraslan et al., 2019). As overcoming the deficiency of causal relationship explanation when only applying GWAS, a framework based on DL was certified to success in identifying causal variants by computing the causal variants in trait-associated loci (Zhou et al., 2018). Another study was conducted to prioritize the IBD-risk genes for detecting a new candidate in IBD-associated genes using an ML-based method. This method used numerical expression data (microarray and RNA-seq) and categorical terms from several databases to categorize genes as risk-conferring or not conferring risk to IBD. It is noteworthy that positive gene instances were collected from GWAS (Isakov et al., 2017). Finally, 67 genes that had never been mentioned in other publications of IBD were identified as a novel candidate in IBD-risk genes. These genes can be used as targets in the future. This study shows that ML can remedy some inherent limitations of GWAS and can analyze the GWAS data.

In another study, investigators attempted to find genes that differentiate patients with IBD from normal individuals and identify their subtypes. After selecting IBD-related genes using the method of minimum redundancy, maximum relevance (mRMR), and incremental feature selection (IFS), the investigators applied protein–protein interaction to establish a network and then applied the shortest path method to obtain other related genes (Yuan et al., 2017). The mRMR is a method for identifying SNPs that provide an mRMR feature list to inform the evaluation of relevance between feature and sample class labels and the redundancies between it and the features listed before it (Chen et al., 2016). Then, the IFS method selects the optimal combination as biomarkers applying the mRMR feature list and the sequential minimal optimization prediction engine (Peng et al., 2005). In the future, phenotypic and clinical data may be used as the basis for selecting an optimal combination, thereby linking genes to more intuitive information and improving the interpretability of data to some extent.

### Relevance Between Extra-Intestinal Manifestations and Genetic Factors

In addition to common digestive symptoms of IBD, extra-intestinal manifestations are also a significant factor affecting the health and quality of life of the patient (Bernstein et al., 2019). In recent years, studies have predicted the possibility of extra-intestinal symptoms in patients with IBD to intervene as early as possible and reduce patient suffering (Herzog et al., 2018). After ML emerged as an efficient approach, these data-based studies were reconsidered. Two studies based on the same data of 152 patients were observed and compared for the efficiencies of NB, Bayesian Additive Regression Trees (BART), and Bayesian Networks (BNs) (Menti et al., 2016; Bottigliengo et al., 2019).

Naïve Bayes is a classifier based on Bayes’ theorem, assuming that there is no interaction between the features except the independent given class that shows outstanding performance in automatic medical diagnosis (Menti et al., 2016). BART is a method using a sum of many regressions or classification trees plus a random component to specify the relationship between the outcome and a set of covariates (Bottigliengo et al., 2019). BNs can also give the probability of event occurrence through computing the conditional probabilities of the parameters in models given the values of variables (Bottigliengo et al., 2019).

In 2016, the identification ability of three Bayesian machine-learning technologies (BMLTs) was compared based on disease characteristics, risk factors, and genetic polymers of the NOD2, CD14, TNF-α, IL12B, and IL1RN genes. Finally, BNs were confirmed to make an identification without a large sample, with 82% (considering only clinical factors) and 89% (considering genetic information as well) accuracies (Menti et al., 2016). They also built an interpretable graphical model that displayed the links between variables and can be modified according to medical knowledge; thus, interdisciplinary research in IBD has its advantages (Menti et al., 2016).

The objective of this study in 2019 was to compare these three BMLTs with logistic regression, generalized additive model, projection pursuit regression (PPR), linear discriminant analysis (LDA), quadratic discriminant analysis, and ANNs taken from the study of Giachino et al. (2007). In this study, BMLTs did not show superiority over the traditional method as expected. Three BMLTs ameliorated the function after considering genetic variables. However, the best performance of three BMLTs was made by BART with 0.76 area under the receiver operating characteristic curve (AUC), which was still worse than PPR (AUC = 0.94). This outcome was caused not only by the method itself but also by the different results of 10-fold cross-validation by evaluation under the premise of selecting different features and folds (Bottigliengo et al., 2019). This reminds us that the data-based approach to ML has a characteristic similar to statistical methods and that the existence of an outlier may introduce a gap in the results.

### Influence of Environmental Factors on IBD Flares

At present, many researchers consider that the imbalance between proinflammatory and anti-inflammatory forces that lead to an occurrence of IBD is a response to genetic and environmental factors (Sears and Johnston, 2007). Several ML models such as ANN and SVM are sufficient for working with complex datasets to compensate for the deficiency of traditional linear statistics (Liu et al., 2013). A study of the association between seasonal changes and onset and recurrence of IBD revealed a high incidence of CD in July and August (Peng et al., 2015). This study used an ANN model to predict the frequency of onset, relapse, and severity of IBD and achieved a high accuracy in predicting the frequency of the relapse of IBD (mean square error = 0.009 and mean absolute percentage error = 17.1%). The previous studies applying multiple logistic regression or multiple LDA models came to conflicting conclusions about the relationship between IBD flares and season variation due to inadequate statistical stability (Peng et al., 2015). Thus, this study confirmed the feasibility of applying ANN methods to verify the correlation between certain factors and disease incidence (Peng et al., 2015; Table 1). Epidemiological studies that directly link risk factors to the incidence of IBD are still instrumental. In addition to suggesting that the association between the two factors provides advice for public health management, the studies may suggest a non-immune, directly related IBD etiological mechanism, including smoking, obesity, and dietary factors such as high-fat or low-fiber diets (Singh et al., 2017).

**TABLE 1:** Summary of studies using artificial intelligence in IBD etiology.

References	Published year	Aim of study	Type of AI	Number of subjects	Input variables (number/type)	Outcomes
Isakov et al., 2017	2017	Prioritization of IBD risk genes to detect candidate novel IBD-associated genes	Four different machine-learning classification models: rf, svmPoly, xgbTree, and glmnet	180 CD, 149 UC, 94 colorectal neoplasms, and 90 normal tissue	309/expression data from both array and RNA-seq data sets, GO, KEGG, and the Pathway Interactions Database terms	67 novel candidate IBD-risk genes
Yuan et al., 2017	2017	Screening for differential expressing genes among different clusters based on an IBD database to identify genes related to IBD	SMO	59 CD, 26 UC, and 42 normal samples	12,754/expression levels of 12,754 genes	21 candidate genes related to IBD
Menti et al., 2016	2016	Assessment of the predictive power of three BMLTs as classifiers for EIM in CD patients	3 BMLTs: NB, BART, and BN	152 patients with CD	12/disease characteristics, risk factors, and genetic variables	Accuracy: 89% (BN achieved the best performance)
Bottigliengo et al., 2019	2019	Determination of whether BMLT could improve EIM prediction	3 BMLTs: NB, BART, and BN	152 patients with CD	12/disease characteristics, risk factors, and genetic variables	Sensitivity: 66.0%, specificity: 69.0% (BART achieved the best performance)
Peng et al., 2015	2015	Prediction of IBD onset and relapse frequency with meteorological data	ANN	569 UC and 332 CD patients	5/meteorological data	Accuracy in predicting the frequency of IBD relapse (mean square error = 0.009, mean absolute percentage error = 17.1%)

*IBD, inflammatory bowel disease; CD, Crohn’s disease; UC, ulcerative colitis; GO, gene ontology; KEGG, Kyoto encyclopedia of genes and genomes; rf, random forest; svmPoly, support vector machine with polynomial kernel; xgbTree, extreme gradient boosting; glmnet, elastic net regularized generalized linear model; SMO, sequential minimal optimization; BMLTs, Bayesian Machine Learning Techniques; EIM, extra-intestinal manifestations; NB, Naïve Bayes; BART: Bayesian Additive Regression Trees; BN, Bayesian Networks; ANN, artificial neural network.*

## Artificial Intelligence in Diagnosis of IBD

In recent years, with the increasing incidence of IBD, seeking more accurate diagnostic tools for IBD has become a hot topic. Currently, there is no gold standard for diagnosing IBD (Annese et al., 2013; Magro et al., 2013). A comprehensive analysis combining clinical presentation, laboratory examination, imaging, endoscopy, and histopathology and exclusion of a range of infectious diseases and intestinal TB is required to provide further treatment plans (Annese et al., 2013; Magro et al., 2013). However, integration of information is influenced by the subjective factors of investigators (Daperno et al., 2017; Bernstein et al., 2019). A survey involving 58 gastroenterologists, using the Mayo endoscopic subscale to evaluate the mucosal healing of patients with UC and Rutgeerts score to evaluate the postoperative scores of patients with CD, showed that the inter-judgment consistency was only 0.47 and 0.33, respectively (Fernandes et al., 2018). This study did not aim to reveal differences between diagnoses in order to emphasize the requirements of the expertise of physicians. Instead, it expressed the need for a more effective IBD scoring system and suggested the feasibility of a “training program” for diagnosis (Fernandes et al., 2018). Based on the characteristics that the diagnosis of IBD relies on the personal experience of physicians and consideration of the defect of personal subjective factors (Annese et al., 2013; Daperno et al., 2017), the application of the diagnosis of ML-assisted IBD shows great potential.

### Image Analysis and Automatic Preprocessing of Images

Some computer-aided diagnosis (CAD) programs combine computers and new image acquisition devices such as endocytoscopes to collect images with higher resolution and then analyze the images using AI, facilitating more accurate and faster diagnoses (Rath et al., 2015).

Convolutional neural networks show excellent performance in image recognition (Abadi et al., 2016). Good results were also demonstrated when applying CAD systems for intestinal diseases based on CNNs (Wimmer et al., 2019). However, there are many deficiencies in this new method, including differences in evaluation caused by the location of image selection and the difficulty in scoring caused by local treatment. These problems are still challenging to eliminate as in traditional imaging studies (Leggett and Wang, 2016; Mori et al., 2017), and the multifactor analysis function of ML technology itself is not fully played.

**FIGURE 3:**
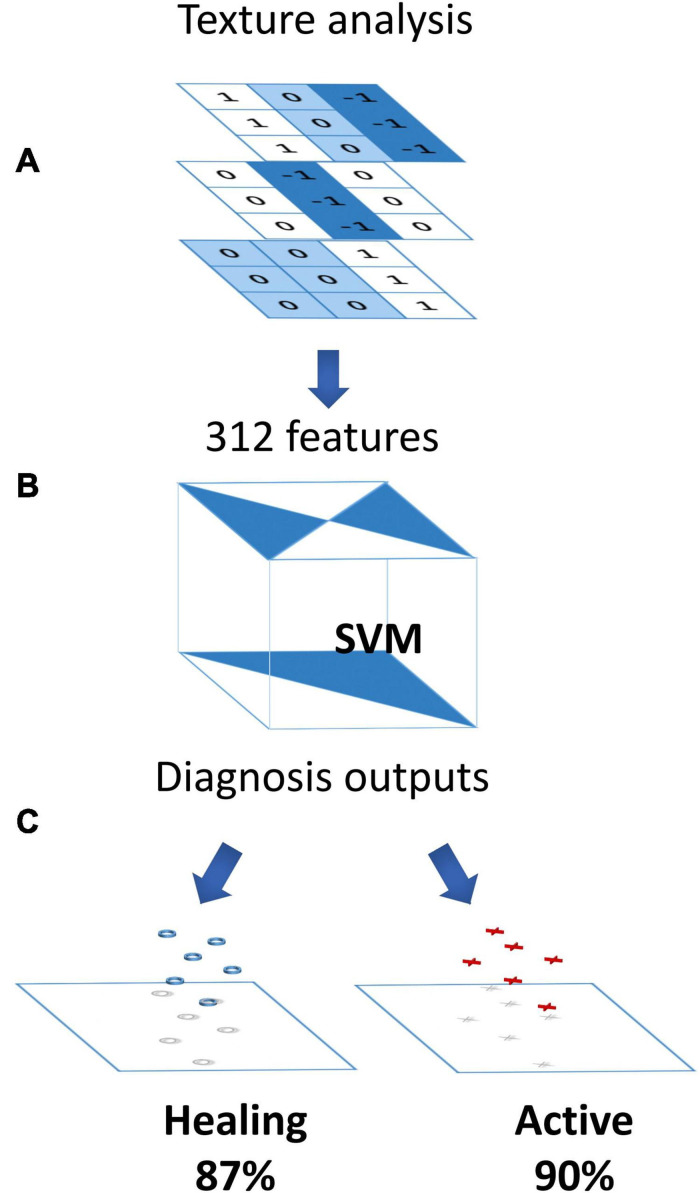
Computer-aided diagnosis for endocytoscopy to identify histological inflammation. **(A)** Three-hundred and twelve features collected by endocytoscopic and texture analysis. **(B)** SVM based on the 312 features classifying 2-class diagnoses (“active” or “healing”). **(C)** The status and diagnostic probability were published. Adapted with permission from Maeda et al. (2019), Elsevier.

A study to improve the diagnosis of IBD by improving a CAD system to analyze the endoscopic images revealed that in addition to achieving higher sensitivity, specificity, and accuracy, the CAD systems have some advantages over traditional imaging such as providing more comprehensive imaging information, implementing an automatic selection of the region of interest, and better reproducibility. Based on the data from 187 patients with UC, a study collected 525 validation sets from 100 patients and utilized the 12,900 endoscopic images of the remaining 87 patients to develop the CAD system. All endoscopic images were marked with their histological assessment according to the biopsy samples. Finally, this CAD system constructed by 312 features on the endoscopic image showed its potential to fully automated the identification of histological inflammation related to UC with 74% diagnostic sensitivity, 97% specificity, and 91% accuracy (Figure 3; Maeda et al., 2019).

Moreover, one study concentrated on the evaluation of endoscopic images in UC, which uses the red density (RD) algorithm refined by integrating computerized vessel pattern recognition and multiple regression analysis. The endoscopic images and biopsy samples from 29 consecutive patients with UC and 6 healthy controls were used to build the algorithm. This algorithm was developed based on automatic computer-aided assessment of redness on a pixel level and was further refined through recalibrating and expanding the RD score with the information of clinical, endoscopic, and histological scorings. Its stable and reproducible outcome conforms with the requirement of objectivity for the diagnosis. The strong correlation between the way how the entire image is digitized and histology suggests its potential for predicting the prognosis (Bossuyt et al., 2018, 2020).

After more ML structural frameworks have been published, the cross-discipline boom has resulted in the broader use of image recognition for the diagnosis of IBD. The future value of CAD and the use of images for data extraction and analysis are very compelling (Leggett and Wang, 2016; Mori et al., 2017). However, some features of medical imaging also show adverse effects on ML applications. The differences in annotation and endoscopic imaging performance result in significant differences in the types of medical image databases and between different databases. Cleaning and preparation of image data have thus become a meaningful and challenging task (Nadeem et al., 2018). Researchers have developed a framework for automatic preprocessing of gastrointestinal tract images (MAPGI) designed to preprocess gastrointestinal images. This framework enables deep neural network recognition of images with better performance through edge removal, contrast enhancement, filtering, color mapping, and scaling (Cogan et al., 2019). In addition to improvements in image processing and improvements to specific ML methods selected are also underway. An attempt to combine the semi-supervised classification with active learning can reduce the amounts of labeled samples required for program training and reduce the time required for training (Mahapatra et al., 2016).

### Machine Learning Enhancing Non-invasive Test for IBD With miRNA Signatures

In addition to the progress made in the diagnosis of IBD imaging and by the influence of technology and other published results, some new methods of assisted diagnosis have been applied. A study uses penalized SVMs and RF for analyzing peripheral blood miRNAs in subjects to achieve a differential diagnosis of CD and UC. It established a sparse model based on the expression levels of certain miRNAs from 76 patients with IBD and 38 healthy controls and then selected 16 distinct miRNAs to be the markers for diagnostic application. Then, the classification was achieved by penalized SVMs and used RF to validate the SVM-based miRNA signatures (Hubenthal et al., 2015). In this study, the elastic SCAD SVM (median AUC = 0.97) was chosen for further investigation and finally achieved the distinction of CD and UC as well as CD, UC, and healthy controls with significantly small classification error rates of 3.1 and 3.3% (Hubenthal et al., 2015).

There are also studies directly analyzing the genes of subjects, trying to classify healthy people and patients with CD according to their genomic information. These studies are different from those simply looking for risk genes, and their purpose is to diagnose patients with clinical signs. In one study, three ML methods, namely, penalized logistic regression, gradient boosted trees, and ANNs, were used to reanalyze the Immunochip dataset genotyped by the International Inflammatory Bowel Disease Genetics Consortium (IIBDGC). This study included 18,227 patients with CD and 34,050 healthy controls, finally resulting in a maximum AUC of 0.80 achieved by LR methods (Romagnoni et al., 2019). Another study integrated predicted pathogenicity, disease gene annotation, and functional interaction network with exome sequence data into a matrix factorization-based ML model and succeeded in the identification of patients with CD with an AUC of 0.816, which also showed the potential of ML methods for drawing disease-associated sequence variants (Jeong and Kim, 2016).

### Determining IBD Subtype and Evaluation of Disease Status

Some studies have made new claims in addition to the diagnosis of IBD. They have tried to classify and stage IBD using ML technology, which is vital for the choice of follow-up therapy.

Plevy et al. (2013) used the information including serology and genetic markers to develop a multicomponent ML model, which has an excellent recognition function for an adult with CD and UC. They constructed a diagnostic RF algorithm based on patient blood samples from 572 cases with CD, 328 cases with UC, 437 controls with non-IBD, and 183 healthy controls, which achieved both identification of patients with IBD and differentiation of patients with CD and UC. The diagnostic accuracy was compared between using a panel of six serological markers only and using a marker panel that in addition to the serological markers, also included four genetic markers, five inflammatory markers, and two additional serological markers during receiver operating characteristic analysis. As expected, with the extension of the marker panel, the area under the curve of the IBD vs. non-IBD discrimination increased from 0.80 to 0.87 as that for the CD vs. UC increased from 0.78 to 0.93 (Plevy et al., 2013).

**FIGURE 4:**
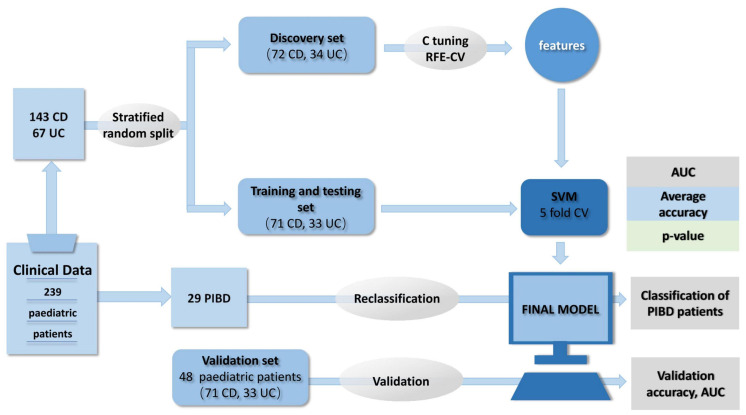
Model construction and data processing. The whole project was composed of model construction, validation, and inflammatory bowel disease unclassified reclassification. The study recruited 239 pediatric patients from the Genetics of Pediatric Inflammatory Bowel Disease study at Southampton Children’s Hospital. The clinical data were used to search the best parameters for classification and train the model. The linear SVM was applied to construct the optimal model, allowing for assessing the relevance with the disease of the selected variable. Then, the optimal penalty parameter (C) tuning and fivefold cross-validation scheme (RFE-CV) help maximizing the classification accuracy by avoiding overfitting. Adapted with permission from Mossotto et al. (2017), Nature Publishing Group.

Mossotto et al. (2017) also developed three supervised ML models based on SVM, utilizing endoscopic data only, histological only, and combined endoscopic/histological data, respectively, to classify pediatric inflammatory bowel disease. Their results proved that the linked model performed better than single histological or endoscopic model with a diagnostic accuracy of 82.7%. Although there was a limitation that the whole study was based on pediatric patients when considering the clinical application and promotion to the broader population, the significance of this study cannot be ignored. They also constructed an unsupervised model and exhibited an overlap of CD/UC with broad clustering but no clear subtype delineation, which was then classified into four distinct groups according to different colorectal involvements (Figure 4; Mossotto et al., 2017). This method has been successfully applied in cancer typing (Upstill-Goddard et al., 2013). This universality is instructive for diseases such as IBD whose etiology is still unclear and has many predisposing factors.

One study attempted to use intestinal microecology to diagnose IBD, with combined human genetic data and 16S and metagenomic (MGS) intestinal biopsy data, to classify patients with CD according to disease status and treatment response (Douglas et al., 2018). From the perspective of IBD etiology, it was efficient to apply information related to intestinal microecology in this study (Sokol and Seksik, 2010). The study based on 40 intestinal biopsy samples used RF to determine classification accuracy when the 16S ribosomal RNA gene (16S) and shotgun MGS collapsed to different hierarchical groupings were used separately to classify patients by disease progression and by the response of patients with CD to treatment (Douglas et al., 2018). As the first study to incorporate human genetic data with 16S and MGS intestinal biopsy data, this study achieved the classification of patients with CD according to disease state and treatment response and concluded the genera identified from 16S data as the best classifiers (Douglas et al., 2018; Table 2). Another study based on 16S rRNA sequencing of the gut microbiome with longitudinal analyzes was performed to develop a model that can determine the dynamics of intestinal flora and the best IBD predictive features. A supervised learning RF model was applied to predict IBD subtypes during the evaluation of the microbiome using predictive tools. This model successfully discriminated IBD subtypes from healthy controls and correctly predicted 66.6% of the samples (66). Compared with other studies gaining analogous accuracy, this study applied accessible fecal samples instead of rectal samples (Gevers et al., 2014).

**TABLE 2:** Summary of studies using artificial intelligence in IBD diagnosis.

References	Published year	Aim of study	Type of AI	Number of subjects	Outcomes
Maeda et al., 2019	2019	Prediction of persistent histologic inflammation associated with UC	SVM	Training set: 12,900 images from 87 patients. Test set: 9935 images from 100 patients	Sensitivity: 74%, specificity: 97%, accuracy: 91%
Mahapatra et al., 2016	2016	Segmentation of CD from abdominal MRI	Active learning framework combined with semi-supervised learning	70 patients (fivefold cross validation)	Dice Metric: 92.4%, Hausdorff distance: 7.0 mm
Hubenthal et al., 2015	2015	Diagnostics of IBD	SVM	114 patients	AUROC: 0.75–1.00
Mossotto et al., 2017	2017	Classification of Pediatric Inflammatory Bowel Disease	SVM	239 patients	Accuracy: 82.7% (model utilizing combined endoscopic/histological data achieved the best performance)
Douglas et al., 2018	2018	Classification of disease state and treatment outcome in pediatric Crohn’s disease	RF	Intestinal biopsies of 20 treatment-naïve CD and 20 control pediatric patients	Accuracy: 84.2% (model utilizing 16S taxonomic datasets achieved the best performance)

*IBD, inflammatory bowel disease; UC, ulcerative colitis; SVM, support vector machine; CD, Crohn’s disease; MRI, magnetic resonance images; AUROC, area under receiver operating characteristic; RF, random forest.*

## Artificial Intelligence in Treatment of IBD

Currently, the goal of treatment of IBD has changed from the traditional clinical remission to a more specific, integrated, and complete deep remission or mucosal healing (Panaccione et al., 2013; Sandborn et al., 2014; Klenske et al., 2019). The advent of new biologics and small molecules such as anti-TNF agents has significantly changed the treatment strategies of IBD. With a better understanding of the IBD pathophysiology, new treatment options including anti-cytokine agents, anti-adhesion molecules, fecal microbiota transplantation, and mesenchymal stem cell therapy become increasingly available (Verstockt et al., 2018). Clinical decisions are more difficult for not only patients but also clinicians. At present, the action targets and predictable tolerability of novel treatment options usually serve as the driving factors for clinical decisions. However, many difficulties remain to be solved in optimizing treatment strategies, improving long-term prognosis, and changing the natural history of IBD (Atreya and Neurath, 2018). ML is thus feasible because of its ability to extract information from existing medical records and digital images for predicting the progression of IBD or the efficacy of certain medications.

### Machine Learning Supporting the Prediction of IBD Progression

One study applied RF algorithms for predicting the likelihood of patients with IBD experiencing disease flares over a certain period. Corticosteroid use and hospitalizations were considered as a surrogate for IBD flares in this study. This study initially limited the predictors, including age, sex, and five other features, for the diagnosis of IBD and used these finite factors to establish models for predicting the IBD flares. Finally, the best prediction performance was achieved by the RF longitudinal model anticipated with previous hospitalization or steroid use, and its AUC reached 0.87 (Waljee et al., 2017). Another study also proved the significant prediction ability of ML. The gradient boosting machines built with information from routinely collected electronic medical records reached a very high accuracy (AUC = 0.93) when predicting the inflammation severity in patients with CD. The authors concluded the baseline laboratory parameters, patient demographic characteristics, and disease location as the most robust predictors (Reddy et al., 2019). A recent study indicated that the accuracy of elafin for revealing strictures in patients with CD was increased with a two-class decision forest algorithm (Wang et al., 2020). These studies showed the application of longitudinal data in predicting disease progression, which was followed by the use of ML methods to predict the prognosis of disease for avoiding over-treatment or delayed treatment.

### Forecasting the Efficacy After Clinical Intervention

In addition to predicting the likelihood of IBD flare emergence, prediction of the possible efficacy of some treatments is also a major direction of further research. A retrospective study analyzed some known patients with a good response to steroids, cyclosporine, or infliximab and selected nine miRNAs plus five clinical factors as predictors of first-line and second-line treatment effects with the help of a deep network-based classifier. The classification accuracy of this panel to discriminate responders to steroids from non-responders reached 93%. Interestingly, a linear program is the most effective way when the number of parameters is increased (Morilla et al., 2019). This study shows the great value of ML technology in feature screening and suggests that traditional statistical methods cannot be ignored in revealing relevance. In another study, the RF algorithm also showed advantages in building complex models and exposing non-linear relationships. The study used the C-creative protein (CRP) level as an evaluation criterion to predict whether patients with moderate to severe CD could be relieved after ustekinumab treatment. The model using data through week 8 had a mean area under the AUC of 0.78. It showed a more remarkable similarity with the previous study, but the selected predictors focused more on clinical and laboratory information such as CRP level and the ratio of serum ustekinumab level to CRP level at several points (Waljee et al., 2019; Table 3).

**TABLE 3:** Summary of studies using artificial intelligence in IBD treatment.

References	Published year	Aim of study	Type of AI	Number of subjects	Input variables (number/type)	Outcomes
Waljee et al., 2017	2017	Prediction of IBD flares	RF	20,368 patients	6/demographic data, lab data, and clinical variables	AUROC: 0.87 (RF longitudinal model utilizing previous hospitalization or steroid use achieved the best performance)
Reddy et al., 2019	2019	Prediction and explanation of inflammation in CD	Gradient boosting machines	82 patients	40/demographic data, lab data, and clinical variables	AUROC: 0.93
Wang et al., 2020	2020	Detection of intestinal strictures in CD patients	Two-class decision forest algorithm	67 patients	15/serum elafin level and 14 clinical variables	AUROC: 0.92 (model utilizing serum elafin levels and commonly available clinical data achieved the best performance)
Morilla et al., 2019	2019	Prediction of responses to therapy of patients with Acute Severe UC	Neural networks	47 patients	14/9 microRNAs and five clinical variables	Accuracy: 93%, AUROC: 0.91
Waljee et al., 2019	2019	Identification of CD patients likely to be durable responders to ustekinumab	RF	401 patients	12/5 demographic data and seven laboratory test results	AUROC: 0.78 (model utilizing data through week 8 achieved the best performance)
Cushing et al., 2019	2019	Prediction of CD recurrence risk after ileocolic resection	RF	60 patients (five patients with extreme variability on whole transcriptome analysis had been excluded)	30/expression levels of 30 transcripts	Accuracy: 91.67% (in anti-TNFα-naïve patients), 92.86% (in patients receiving anti-TNFα therapy)
Jones et al., 2020	2020	Prediction of sustained remission after exclusive enteral nutrition in pediatric CD	RF	22 patients	34/demographic data, clinical variables, and 27 microbial data	AUROC: 0.9 (model utilizing microbial abundances, species richness, and Paris disease classification achieved the best performance)

*IBD, inflammatory bowel disease; CD, Crohn’s disease; UC, ulcerative colitis; AUROC, area under receiver operating characteristic; RF, random forest.*

The ileocolic resection is an essential treatment for a large number of patients with CD after a long disease progression, and the rate of the second or third surgeries gets to 23–43% (Cushing et al., 2019). The prediction of postoperative disease recurrence risk is helpful for clinical decisions and has been proved viable by a study resorting RF algorithm to analyze the whole transcriptome array from 65 patients (Cushing et al., 2019). And 30 influential transcripts in anti-TNF-naive patients uncovered in this study can be the reference for anti-TNF efficacy studies.

To date, with a better understanding of how gut microbiota participates in IBD pathogenesis, there are more and more studies focusing on its predictive value. Casey et al. studied the gut microbiome taxonomy and functional capacity in 139 fecal samples of 22 patients (8–15 years of age) with CD who underwent treatment with exclusive enteral nutrition (EEN). The RF model was employed to classify treatment response and successfully made a distinction between sustained remission and those who neither achieved remission nor relapsed by 24 weeks with clinical metadata and baseline microbiome. The microbiome data and Paris classification of disease behavior and location were confirmed as the best feature for prediction, which may imply the clinical application indicator of EEN (Jones et al., 2020).

### Machine Learning Facilitating Personalized Therapeutic Intervention

A study on the use of ML technology to achieve precision medicine is also underway. The multifactor analysis capabilities of ML facilitate the provision of personalized therapies (Williams et al., 2018). A study on pediatric IBD treatment considering the heterogeneity of IBD proposed a plan to comprehensively analyze genetic factors, environmental factors, and combine clinical data and laboratory information to develop a drug treatment project (Ashton and Beattie, 2019). Nowadays, with the emergence of novel therapies in IBD, the demand for effective disease management becomes more insistent. Then, the potential of ML to provide personalized predictions becomes more concerned because these methods improve the prognosis of patients and reduce healthcare expenditure (Williams et al., 2018).

## Discussion

In the entire field of gastroenterology, AI is mainly used in image recognition and statistical analysis of diagnosis or prediction of prognosis (Yang and Bang, 2019). From past studies, CNN has shown excellent accuracy in image recognition, and its growth was confirmed by the outstanding performance at the ImageNet Large Visual Recognition Competition (ILSVRC) (Yang and Bang, 2019). For IBD, fewer studies than expected simply aim to use diagnosis or staging models based on image analysis to eliminate inter-observer differences. In comparison, image standardization procedures as the auxiliary tool for image analysis seem to be a highlight raising attention as image preprocessing operations of cropping and contrast enhancement are essential for further research (Cogan et al., 2019).

At present, natural language processing as the important area of AI could bring another peak of AI application in IBD research based on clinical data due to its ability to extract information from plain text (Wu et al., 2020). In the basic research, system biology serves as a holistic approach to elucidate the complexity of pathophysiological mechanisms of disease in a cross-disciplinary environment. The automatic feature learning ability of ML algorithms can enhance the feature selection in system biology approaches, which demand manual programming all along. A large number of existing studies concentrate on AI applications for genome analysis (Zou et al., 2019). However, the evolvement of transcriptomics, proteomics, and metabolomics can be further accelerated with the support of analytical approaches such as ML. When considering that precision medicine involves accurate diagnosis, credible risk assessment, and individual treatment, AI seems to be a technique that can boost its development and also benefit from its broad-scale application. Longitudinal study data could be the basis for the utilization of these advanced analytical methods in all these three IBD management stages (Ashton and Beattie, 2019). There is still more feasibility for AI-assisted IBD studies to promote the constitution of translational precision medicine.

In addition to the advantages of AI technology, there are still some problems that cannot be ignored. AI technology has inherent pitfalls, such as overfitting, selection bias, and spectrum bias (class imbalance), which may lead to overestimation of accuracy or inappropriate generalization of results (England and Cheng, 2019). External validation using vacant datasets collected in a manner that minimizes the spectrum bias and prospective studies with competent inclusion/exclusion criteria that represent the target population is needed (Yang and Bang, 2019). However, there are more points than academic effectiveness to consider the performance of AI-assisted IBD studies. The high cost of some tests buried in their novelty should be an indicative competent to judge their desirability from the aspect of translational medicine. Further investigation is essential for maximizing the performance of applying AI technology in IBD studies and understanding its feasibility.

## Search Strategy and Selection Criteria

Articles for this study were identified using PubMed, and references from relevant articles using the following search terms: “Inflammatory bowel disease” OR “Crohn’s disease” OR “Ulcerative colitis,” AND “Artificial intelligence” OR “Machine learning” OR “Artificial neural network” OR “Convolutional neural network.” Only the most impactful papers were considered.

## Conclusion

Artificial intelligence can boost studies on IBD by identifying the susceptibility loci and other IBD etiological factors. For the diagnosis of IBD, the images and miRNA signatures can be analyzed by AI, and predicting flares are effective for treatment.

## Author Contributions

GC collected the papers and data, analyzed the conclusions, and drafted the manuscript. JS presented the idea of this manuscript, supported the funding, analyzed the conclusions, and drafted and revised the manuscript. Both authors contributed to the article and approved the submitted version.

## Conflict of Interest

The authors declare that the research was conducted in the absence of any commercial or financial relationships that could be construed as a potential conflict of interest.

## References

[B1] Abadi M. Agarwal A. Barham P. Brevdo E. Zheng X. (2016). *TensorFlow: Large-Scale Machine Learning on Heterogeneous Distributed Systems. arXiv [Preprint].* Available online at: https://arxiv.org/abs/1603.04467 ^∗^accq.

[B2] Annese V. Daperno M. Rutter M. D. Amiot A. Bossuyt P. East J. (2013). European evidence based consensus for endoscopy in inflammatory bowel disease. *J. Crohns Colitis* 7 982–1018. 24184171 10.1016/j.crohns.2013.09.016

[B3] Ashton J. J. Beattie R. M. J. L. (2019). Personalised therapy for inflammatory bowel disease. *Lancet* 393 1672–1674. 10.1016/s0140-6736(18)33125-8 30935733

[B4] Atreya R. Neurath M. F. (2018). Mechanisms of molecular resistance and predictors of response to biological therapy in inflammatory bowel disease. *Lancet Gastroenterol. Hepatol.* 3 790–802. 10.1016/s2468-1253(18)30265-6 30353856

[B5] Bernstein C. N. Benchimol E. I. Bitton A. Murthy S. K. Nguyen G. C. Lee K. (2019). The impact of inflammatory bowel disease in Canada 2018: extra-intestinal diseases in IBD. *J. Can. Assoc. Gastroenterol.* 2 S73–S80. 31294387 10.1093/jcag/gwy053 PMC6512250

[B6] Bossuyt P. Nakase H. Vermeire S. De Hertogh G. Eelbode T. Ferrante M. (2020). Automatic, computer-aided determination of endoscopic and histological inflammation in patients with mild to moderate ulcerative colitis based on red density. *Gut* 69 1778–1786. 10.1136/gutjnl-2019-320056 31915237

[B7] Bossuyt P. Nakase H. Vermeire S. Willekens H. Ikemoto Y. Makino T. (2018). 436 - Automated digital calculation of endoscopic inflammation in ulcerative colitis: results of the red density study. *Gastroenterology* 154 S–98–S–99. 10.1136/gutjnl-2019-320056 31915237

[B8] Bottigliengo D. Berchialla P. Lanera C. Azzolina D. Lorenzoni G. Martinato M. (2019). The role of genetic factors in characterizing extra-intestinal manifestations in crohn’s disease patients: are bayesian machine learning methods improving outcome predictions? *J. Clin. Med.* 8:865. 10.3390/jcm8060865 31212952 PMC6617350

[B9] Chen L. Chu C. Feng K. (2016). Predicting the types of metabolic pathway of compounds using molecular fragments and sequential minimal optimization. *Comb. Chem. High Throughput Screen.* 19 136–143. 10.2174/1386207319666151110122453 26552441

[B10] Cogan T. Cogan M. Tamil L. (2019). MAPGI: accurate identification of anatomical landmarks and diseased tissue in gastrointestinal tract using deep learning. *Comput. Biol. Med.* 111:103351. 10.1016/j.compbiomed.2019.103351 31325742

[B11] Cushing K. C. Mclean R. Mcdonald K. G. Gustafsson J. K. Knoop K. A. Kulkarni D. H. (2019). Predicting risk of postoperative disease recurrence in crohn’s disease: patients with indolent crohn’s disease have distinct whole transcriptome profiles at the time of first surgery. *Inflamm. Bowel Dis.* 25 180–193. 10.1093/ibd/izy228 29982468 PMC6354560

[B12] Daperno M. Comberlato M. Bossa F. Armuzzi A. Biancone L. Bonanomi A. G. (2017). Training programs on endoscopic scoring systems for inflammatory bowel disease lead to a significant increase in interobserver agreement among community gastroenterologists. *J. Crohns Colitis* 11 556–561. 28453758 10.1093/ecco-jcc/jjw181

[B13] Douglas G. M. Hansen R. Jones C. M. A. Dunn K. A. Comeau A. M. Bielawski J. P. (2018). Multi-omics differentially classify disease state and treatment outcome in pediatric Crohn’s disease. *Microbiome* 6:13. 10.1186/s40168-018-0398-3 PMC5769311 29335008

[B14] Du Y. Xie J. Chang W. Han Y. Cao G. (2012). Genome-wide association studies: inherent limitations and future challenges. *Front. Med.* 6:444–450. 10.1007/s11684-012-0225-3 23124883

[B15] England J. R. Cheng P. M. (2019). Artificial intelligence for medical image analysis: a guide for authors and reviewers. *AJR. Am. J. Roentgenol.* 212 513–519. 10.2214/ajr.18.20490 30557049

[B16] Eraslan G. Avsec Ž Gagneur J. Theis F. J. (2019). Deep learning: new computational modelling techniques for genomics. *Nat. Rev. Genet.* 20 389–403. 10.1038/s41576-019-0122-6 30971806

[B17] Fernandes S. R. Pinto J. Marques Da Costa P. Correia L. (2018). Disagreement among gastroenterologists using the mayo and rutgeerts endoscopic scores. *Inflamm. Bowel Dis.* 24 254–260. 10.1093/ibd/izx066 29361106

[B18] Gevers D. Kugathasan S. Denson L. A. Vázquez-Baeza Y. Van Treuren W. Ren B. (2014). Microbe the treatment-naive microbiome in new-onset crohn’s disease. *Cell Host Microbe* 15 382–392. 24629344 10.1016/j.chom.2014.02.005 PMC4059512

[B19] Giachino D. F. Regazzoni S. Bardessono M. Marchi M. D. Gregori D. J. (2007). Modeling the role of genetic factors in characterizing extra-intestinal manifestations in Crohn’s disease patients: does this improve outcome predictions? *Curr. Med. Res. Opin.* 23 1657–1665. 10.1185/030079907x210471 17588296

[B20] Herzog D. Fournier N. Buehr P. Rueger V. Koller R. Heyland K. (2018). Age at disease onset of inflammatory bowel disease is associated with later extraintestinal manifestations and complications. *Eur. J. Gastroenterol. Hepatol.* 30 598–607. 10.1097/meg.0000000000001072 29360691

[B21] Hubenthal M. Hemmrich-Stanisak G. Degenhardt F. Szymczak S. Du Z. Elsharawy A. (2015). Sparse modeling reveals miRNA signatures for diagnostics of inflammatory bowel disease. *PLoS One* 10:e0140155. 10.1371/journal.pone.0140155 26466382 PMC4605644

[B22] Isakov O. Dotan I. Ben-Shachar S. (2017). Machine learning-based gene prioritization identifies novel candidate risk genes for inflammatory bowel disease. *Inflamm. Bowel Dis.* 23 1516–1523. 10.1097/mib.0000000000001222 28795970

[B23] Jeong C.-S. Kim D. (2016). Inferring crohn’s disease association from exome sequences by integrating biological knowledge. *BMC Med. Genomics* 9(Suppl. 1):35. 10.1186/s12920-016-0189-2 27535358 PMC4989895

[B24] Johnson K. W. Torres Soto J. Glicksberg B. S. Shameer K. Miotto R. Ali M. (2018). Artificial intelligence in cardiology. *J. Am. Coll. Cardiol.* 71 2668–2679. 29880128 10.1016/j.jacc.2018.03.521

[B25] Jones C. M. A. Connors J. Dunn K. A. Bielawski J. P. Comeau A. M. Langille M. G. I. (2020). Bacterial taxa and functions are predictive of sustained remission following exclusive enteral nutrition in pediatric crohn’s disease. *Inflamm. Bowel Dis.* 26 1026–1037. 10.1093/ibd/izaa001 31961432 PMC7301407

[B26] Jostins L. Ripke S. Weersma R. K. Duerr R. H. Mcgovern D. P. Hui K. Y. (2012). Host-microbe interactions have shaped the genetic architecture of inflammatory bowel disease. *Nature* 491 119–124. 23128233 10.1038/nature11582 PMC3491803

[B27] Khor B. Gardet A. Xavier R. J. (2011). Genetics and pathogenesis of inflammatory bowel disease. *Nature* 474 307–317. 21677747 10.1038/nature10209 PMC3204665

[B28] Klenske E. Bojarski C. Waldner M. Rath T. Neurath M. F. Atreya R. (2019). Targeting mucosal healing in Crohn’s disease: what the clinician needs to know. *Therap. Adv. Gastroenterol.* 12:1756284819856865. 10.1177/1756284819856865 PMC6572879 31236140

[B29] Leggett C. L. Wang K. K. (2016). Computer-aided diagnosis in GI endoscopy: looking into the future. *Gastrointest. Endosc.* 84 842–844. 10.1016/j.gie.2016.07.045 27742045

[B30] Liu J. Z. Van Sommeren S. Huang H. Ng S. C. Alberts R. Takahashi A. (2015). Association analyses identify 38 susceptibility loci for inflammatory bowel disease and highlight shared genetic risk across populations. *Nat. Genet.* 47 979–986. 10.1038/ng.3359 26192919 PMC4881818

[B31] Liu X. Li N.-S. Lv L.-S. Huang J.-H. Tang H. Chen J.-X. (2013). A comparison of the performances of an artificial neural network and a regression model for GFR estimation. *Am. J. Kidney Dis.* 62 1109–1115. 10.1053/j.ajkd.2013.07.010 24011972

[B32] Luo Y. De Lange K. M. Jostins L. Moutsianas L. Randall J. Kennedy N. A. (2017). Exploring the genetic architecture of inflammatory bowel disease by whole-genome sequencing identifies association at ADCY7. *Nat. Genet.* 49 186–192. 10.1038/ng.3761 28067910 PMC5289625

[B33] Maeda Y. Kudo S. E. Mori Y. Misawa M. Ogata N. Sasanuma S. (2019). Fully automated diagnostic system with artificial intelligence using endocytoscopy to identify the presence of histologic inflammation associated with ulcerative colitis (with video). *Gastrointest. Endosc.* 89 408–415. 10.1016/j.gie.2018.09.024 30268542

[B34] Magro F. Langner C. Driessen A. Ensari A. Geboes K. Mantzaris G. J. (2013). European consensus on the histopathology of inflammatory bowel disease. *J. Crohns Colitis* 7 827–851. 23870728 10.1016/j.crohns.2013.06.001

[B35] Mahapatra D. Vos F. M. Buhmann J. M. (2016). Active learning based segmentation of Crohns disease from abdominal MRI. *Comput. Methods Programs Biomed.* 128 75–85. 10.1016/j.cmpb.2016.01.014 27040833

[B36] Menti E. Lanera C. Lorenzoni G. Giachino D. F. Marchi M. Gregori D. (2016). Bayesian machine learning techniques for revealing complex interactions among genetic and clinical factors in association with extra-intestinal manifestations in IBD patients. *AMIA Annu. Symp. Proc.* 2016 884–893. 28269885 PMC5333221

[B37] Momozawa Y. Dmitrieva J. Theatre E. Deffontaine V. Rahmouni S. Charloteaux B. (2018). IBD risk loci are enriched in multigenic regulatory modules encompassing putative causative genes. *Nat. Commun.* 9:2427. 10.1038/s41467-018-04365-8 PMC6013502 29930244

[B38] Mori Y. Kudo S. E. Berzin T. M. Misawa M. Takeda K. (2017). Computer-aided diagnosis for colonoscopy. *Endoscopy* 49 813–819. 10.1055/s-0043-109430 28561195 PMC6193286

[B39] Morilla I. Uzzan M. Laharie D. Cazals-Hatem D. Denost Q. Daniel F. (2019). Colonic MicroRNA profiles, identified by a deep learning algorithm, that predict responses to therapy of patients with acute severe ulcerative colitis. *Clin. Gastroenterol. Hepatol.* 17 905–913. 10.1016/j.cgh.2018.08.068 30223112

[B40] Mossotto E. Ashton J. J. Coelho T. Beattie R. M. Macarthur B. D. Ennis S. (2017). Classification of paediatric inflammatory bowel disease using machine learning. *Sci. Rep.* 7:2427. 10.1038/s41598-017-02606-2 PMC5445076 28546534

[B41] Na S.-Y. Moon W. (2019). Perspectives on current and novel treatments for inflammatory bowel disease. *Gut Liver* 13 604–616. 10.5009/gnl19019 31195433 PMC6860034

[B42] Nadeem S. Tahir M. A. Naqvi S. S. A. Zaid M. (2018). “Ensemble of texture and deep learning features for finding abnormalities in the gastro-intestinal tract,” in *Computational Collective Intelligence. ICCCI 2018. Lecture Notes in Computer Science*, Vol. 11056 eds Nguyen N. Pimenidis E. Khan Z. Trawiński B. (Cham: Springer International Publishing), 469–478. *editmade. 10.1007/978-3-319-98446-9_44

[B43] Negreanu L. Voiosu T. State M. Voiosu A. Bengus A. Mateescu B. R. (2019). Endoscopy in inflammatory bowel disease: from guidelines to real life. *Therap. Adv. Gastroenterol.* 12:1756284819865153. 10.1177/1756284819865153 PMC6657117 31384307

[B44] Nishida A. Inoue R. Inatomi O. Bamba S. Naito Y. Andoh A. (2018). Gut microbiota in the pathogenesis of inflammatory bowel disease. *Clin. J. Gastroenterol.* 11 1–10. 29285689 10.1007/s12328-017-0813-5

[B45] Panaccione R. Colombel J. F. Louis E. Peyrin-Biroulet L. Sandborn W. J. (2013). Evolving definitions of remission in Crohn’s disease. *Inflamm. Bowel Dis.* 19 1645–1653. 23598817 10.1097/MIB.0b013e318283a4b3

[B46] Peng H. Long F. Ding C. (2005). Feature selection based on mutual information: criteria of max-dependency, max-relevance, and min-redundancy. *IEEE Trans. Pattern Anal. Mach. Intell.* 27 1226–1238. 10.1109/tpami.2005.159 16119262

[B47] Peng J. C. Ran Z. H. Shen J. (2015). Seasonal variation in onset and relapse of IBD and a model to predict the frequency of onset, relapse, and severity of IBD based on artificial neural network. *Int. J. Colorectal Dis.* 30 1267–1273. 10.1007/s00384-015-2250-6 25976931

[B48] Piovani D. Danese S. Peyrin-Biroulet L. Nikolopoulos G. K. Lytras T. Bonovas S. (2019). Environmental risk factors for inflammatory bowel diseases: an umbrella review of meta-analyses. *Gastroenterology* 157 647–659.e4. 31014995 10.1053/j.gastro.2019.04.016

[B49] Plevy S. Silverberg M. S. Lockton S. Stockfisch T. Croner L. Stachelski J. (2013). Combined serological, genetic, and inflammatory markers differentiate non-IBD, Crohn’s disease, and ulcerative colitis patients. *Inflamm. Bowel Dis.* 19 1139–1148. 10.1097/mib.0b013e318280b19e 23518807 PMC3792797

[B50] Rath T. Tontini G. E. Neurath M. F. Neumann H. (2015). From the surface to the single cell: novel endoscopic approaches in inflammatory bowel disease. *World J. Gastroenterol.* 21 11260–11272. 10.3748/wjg.v21.i40.11260 26523101 PMC4616203

[B51] Reddy B. K. Delen D. Agrawal R. K. (2019). Predicting and explaining inflammation in Crohn’s disease patients using predictive analytics methods and electronic medical record data. *Health Informatics J.* 25 1201–1218. 10.1177/1460458217751015 29320910

[B52] Romagnoni A. Jegou S. Van Steen K. Wainrib G. Hugot J. P. (2019). Comparative performances of machine learning methods for classifying crohn disease patients using genome-wide genotyping data. *Sci. Rep.* 9:10351. 10.1038/s41598-019-46649-z PMC6637191 31316157

[B53] Sandborn W. J. Hanauer S. Van Assche G. Panes J. Wilson S. Petersson J. (2014). Treating beyond symptoms with a view to improving patient outcomes in inflammatory bowel diseases. *J. Crohns Colitis* 8 927–935. 10.1016/j.crohns.2014.02.021 24713173

[B54] Sears M. R. Johnston N. W. (2007). Understanding the september asthma epidemic. *J. Allergy Clin. Immunol.* 120 526–529. 10.1016/j.jaci.2007.05.047 17658590 PMC7172191

[B55] Singh S. Dulai P. S. Zarrinpar A. Ramamoorthy S. Sandborn W. J. (2017). Obesity in IBD: epidemiology, pathogenesis, disease course and treatment outcomes. *Nat. Rev. Gastroenterol. Hepatol.* 14 110–121. 10.1038/nrgastro.2016.181 27899815 PMC5550405

[B56] Sokol H. Seksik P. (2010). The intestinal microbiota in inflammatory bowel diseases: time to connect with the host. *Curr. Opin. Gastroenterol.* 26 327–331. 10.1097/mog.0b013e328339536b 20445446

[B57] Topol E. J. (2019). High-performance medicine: the convergence of human and artificial intelligence. *Nat. Med.* 25 44–56. 10.1038/s41591-018-0300-7 30617339

[B58] Upstill-Goddard R. Eccles D. Ennis S. Rafiq S. Tapper W. Fliege J. (2013). Support vector machine classifier for estrogen receptor positive and negative early-onset breast cancer. *PLoS One* 8:e68606. 10.1371/journal.pone.0068606 23894323 PMC3716652

[B59] Verstockt B. Ferrante M. Vermeire S. Van Assche G. (2018). New treatment options for inflammatory bowel diseases. *J. Gastroenterol.* 53 585–590. 29556726 10.1007/s00535-018-1449-z PMC5910475

[B60] Waljee A. K. Lipson R. Wiitala W. L. Zhang Y. Liu B. Zhu J. (2017). Predicting hospitalization and outpatient corticosteroid use in inflammatory bowel disease patients using machine learning. *Inflamm. Bowel Dis.* 24 45–53. 10.1093/ibd/izx007 29272474 PMC5931801

[B61] Waljee A. K. Wallace B. I. Cohen-Mekelburg S. Liu Y. Liu B. Sauder K. (2019). Development and validation of machine learning models in prediction of remission in patients with moderate to severe crohn disease. *JAMA Netw. Open* 2:e193721. 10.1001/jamanetworkopen.2019.3721 31074823 PMC6512283

[B62] Wang J. Ortiz C. Fontenot L. Xie Y. Ho W. Mattai S. A. (2020). High circulating elafin levels are associated with Crohn’s disease-associated intestinal strictures. *PLoS One* 15:e0231796. 10.1371/journal.pone.0231796 32287314 PMC7156098

[B63] Williams A. M. Liu Y. Regner K. R. Jotterand F. Liu P. Liang M. (2018). Artificial intelligence, physiological genomics, and precision medicine. *Physiol. Genomics* 50 237–243. 10.1152/physiolgenomics.00119.2017 29373082 PMC5966805

[B64] Wimmer G. Gadermayr M. Wolkersdorfer G. Kwitt R. Tamaki T. Tischendorf J. (2019). Quest for the best endoscopic imaging modality for computer-assisted colonic polyp staging. *World J. Gastroenterol.* 25 1197–1209. 10.3748/wjg.v25.i10.1197 30886503 PMC6421240

[B65] Windsor J. W. Kaplan G. G. (2019). Evolving epidemiology of IBD. *Curr. Gastroenterol. Rep.* 21:40. 10.1007/s11894-019-0705-6 31338613

[B66] Wu S. Roberts K. Datta S. Du J. Ji Z. Si Y. (2020). Deep learning in clinical natural language processing: a methodical review. *J. Am. Med. Inform. Assoc.* 27 457–470. 31794016 10.1093/jamia/ocz200 PMC7025365

[B67] Yang Y. J. Bang C. S. (2019). Application of artificial intelligence in gastroenterology. *World J. Gastroenterol.* 25 1666–1683. 10.3748/wjg.v25.i14.1666 31011253 PMC6465941

[B68] Ye B. D. McGovern D. P. B. (2016). Genetic variation in IBD: progress, clues to pathogenesis and possible clinical utility. *Exp. Rev. Clin. Immunol.* 12 1091–1107. 10.1080/1744666x.2016.1184972 27156530 PMC5083126

[B69] Yuan F. Zhang Y. H. Kong X. Y. Cai Y. D. (2017). Identification of candidate genes related to inflammatory bowel disease using minimum redundancy maximum relevance, incremental feature selection, and the shortest-path approach. *Biomed. Res. Int.* 2017:5741948. 10.1155/2017/5741948 PMC5331171 28293637

[B70] Yue B. Luo X. Yu Z. Mani S. Wang Z. Dou W. (2019). Inflammatory bowel disease: a potential result from the collusion between gut microbiota and mucosal immune system. *Microorganisms* 7:440. 10.3390/microorganisms7100440 31614539 PMC6843348

[B71] Zhou J. Theesfeld C. L. Yao K. Chen K. M. Wong A. K. Troyanskaya O. G. (2018). Deep learning sequence-based ab initio prediction of variant effects on expression and disease risk. *Nat. Genet.* 50 1171–1179. 10.1038/s41588-018-0160-6 30013180 PMC6094955

[B72] Zou J. Huss M. Abid A. Mohammadi P. Torkamani A. Telenti A. (2019). A primer on deep learning in genomics. *Nat. Genet.* 51 12–18. 10.1038/s41588-018-0295-5 30478442 PMC11180539

